# Oncogene-triggered suppression of DNA repair leads to DNA instability in cancer

**DOI:** 10.18632/oncotarget.2259

**Published:** 2014-07-25

**Authors:** Julia A. Yaglom, Christopher McFarland, Leonid Mirny, Michael Y. Sherman

**Affiliations:** ^1^ Department Biochemistry, Boston University School of Medicine, Boston, MA; ^2^ Harvard Graduate Program in Biophysics, Harvard University, Cambridge, MA; ^3^ Institute for Medical Engineering and Sciences, Massachusetts Institute of Technology, Cambridge, MA

**Keywords:** senescence, oncogenes, DNA Damage Response, Her2

## Abstract

DNA instability is an important contributor to cancer development. Previously, defects in the chromosome segregation and excessive DNA double strand breaks due to the replication or oxidative stresses were implicated in DNA instability in cancer. Here, we demonstrate that DNA instability can directly result from the oncogene-induced senescence signaling. Expression of the activated form of Her2 oncogene, NeuT, in immortalized breast epithelial cells led to downregulation of the major DNA repair factor histone H2AX and a number of other components of the HR and NHEJ double strand DNA breaks repair pathways. H2AX expression was regulated at the transcriptional level via a senescence pathway involving p21-mediated regulation of CDK and Rb1. The p21-dependent downregulation of H2AX was seen both in cell culture and the MMTV-neu mouse model of Her2-positive breast cancer. Importantly, downregulation of H2AX upon Her2/NeuT expression impaired repair of double strand DNA breaks. This impairment resulted in both increased DNA instability in the form of somatic copy number alterations, and in increased sensitivity to the chemotherapeutic drug doxorubicin. Overall, these findings indicate that the Her2/NeuT oncogene signaling directly potentiates DNA instability and increases sensitivity to DNA damaging treatments.

## INTRODUCTION

Most cancer cells are characterized by genomic instability manifested by aneuploidy with numerous karyotypic changes including whole chromosome gain and losses, translocations, deletions, amplifications, etc. According to current paradigm in the field, DNA instability is either the root cause of cancer or a critical contributor to cancer development. Genetic instability, i.e. aneuploidy, often results from abnormalities in mitosis [1], as well as abnormalities in the double strand break repair or telomere maintenance that can lead to repeated chromosome breakage–fusion–bridge cycles [2].

DNA instability (somatic copy number alterations, SCNA) is pervasive in tumors of different origin. Overall, it seems that genomic instability occurs early during tumorgenesis. For example, clonal populations within distant metastases from a pancreatic cancer were shown to be represented within the primary carcinoma, indicating that the genetic heterogeneity of metastases can exist before disseminating from the primary carcinoma [3]. Similarly, the degree of loss of heterozygosity (multiple deletions) in Her2-positive cancers [4] paralleled a substantial SCNA in early lesions, e.g. in breast lobular carcinoma in situ [5]. Interestingly, further progression towards cancer was not associated with higher instability. Indeed, amplifications and deletions in ductal carcinoma in situ (DCIS) were similar to those in neighboring tumors [6]. SCNAs in DCIS and invasive carcinoma also exhibit close similarity [7]. Therefore, most DNA instability in Her2-positive breast cancer arises in early lesions. The mechanisms of this instability may result from either oncogene activation or tumor suppressor inactivation. For example, expression of H-RasV12 or inactivation of PTEN or Rb1 induces chromosomal instability in various cell lines [8, 9]. Recently, we linked oncogene-induced senescence (OIS) pathway with downregulation of the major DNA repair factor histone H2AX, and suggested that this pathway may be directly responsible for DNA instability in early lesions [10]. OIS represents an early barrier to neoplastic transformation [11-20], and therefore in order to become cancerous, cells acquire mutations or epigenetic changes that allow them to “turn off” the senescence program.

We have recently demonstrated that the morphological and biochemical signs of senescence are not rigidly linked to permanent growth arrest. In fact, while activation of the oncogenes in epithelial cells with normal finite lifespan causes genuine senescence, activation of Her2/NeuT or Ras oncogenes in immortalized cells expressing endogenous telomerase triggers morphological and biochemical manifestations of senescence (e.g. SA-β-gal), but does not ensure full growth arrest [10]. We called this condition Senescence With Incomplete Growth arrest (SWING). Importantly, SWING cells, despite their senescent appearance, are transformed and support tumor growth in xenographts [10]. Interestingly, in several types of mouse and human cancer proliferating cells in tumors simultaneously showed positive staining for SA-β-gal [19, 21, 22]. Based on these data, we suggested that SWING could be an intermediate step in carcinogenesis *in vivo*.

One of the major properties of cells in the SWING state induced by expression of the Her2 oncogene was downregulation of histone H2AX [10]. This findings lay the basis for a hypothesis that expression of an active Her2 oncogene results in suppression of the DNA repair systems, which drives genetic instability, and here, we test this hypothesis.

## RESULTS

### Her2/NeuT oncogene causes downregulation of H2AX in a p21-dependent manner

**Fig. 1:**
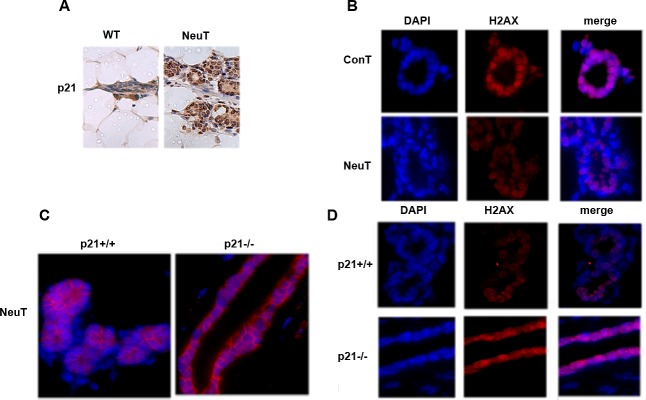
p21 knockout reverses downregulation of H2AX by NeuT expression A. Expression of NeuT downregulates H2AX in 12 months old mice. Control and MMTVneu mice were sacrificed at the age of 12 months and expression of H2AX was assessed in mammary tissue by immunostaining. B. Knockout of p21 does not reduce NeuT expression in MMTVneu model. Mammary glands of 12-months-old NeuT^+/-^p21^+/+^ and NeuT^+/-^p21^-/-^ mice were immunostained for Her2/NeuT. C. p21 knockout restored expression of H2AX in NeuT+/- mice. Mammary glands of 12 months old NeuT^+/-^p21^+/+^ and NeuT^+/-^p21^-/-^ mice were immunostained for H2AX.

Previously we demonstrated that expression of NeuT in immortalized breast cancer epithelial cells MCF10A leads to significant downregulation of the major DNA repair factor histone H2AX. This effect required the upregulation of the senescence mediator p21 (1). Here we investigated if this pathway functions in vivo in the MMTV-neu mouse model of Her2-positive breast cancer. The age of tumor emergence in this strain of mice is approximately 12 month. Accordingly, 12-month-old NeuT-expressing (MMTV-neu) mice and control FVB/NJ female mice were sacrificed, and levels of p21 and H2AX were assessed in isolated mammary tissue by immunohistochemistry with corresponding antibodies. Similar to cell culture experiments and in vivo observations in young (3 months old) mice (1), expression of NeuT caused significant up-regulation of p21 and simultaneously down-regulation of H2AX (Fig. 1A, B). To address if Her2-mediated suppression of H2AX is p21-dependent *in vivo* we crossed NeuT-expressing (MMTV-neu) mice with p21 knockout mice to obtain a NeuT^+/-^p21^-/-^ female litter. NeuT^+/-^p21^+/+^ females were used as a control (see Material and Methods). As seen on Fig. 1C, both strains demonstrated strong expression of NeuT. Importantly, knockout of p21 reversed the downregulation of H2AX in NeuT-expressing mice (Fig. 1D). Therefore, the Her2-p21-H2AX pathway we previously identified in a breast cancer cell line is also functional *in vivo* in mouse mammary tissue.

**Fig. 2:**
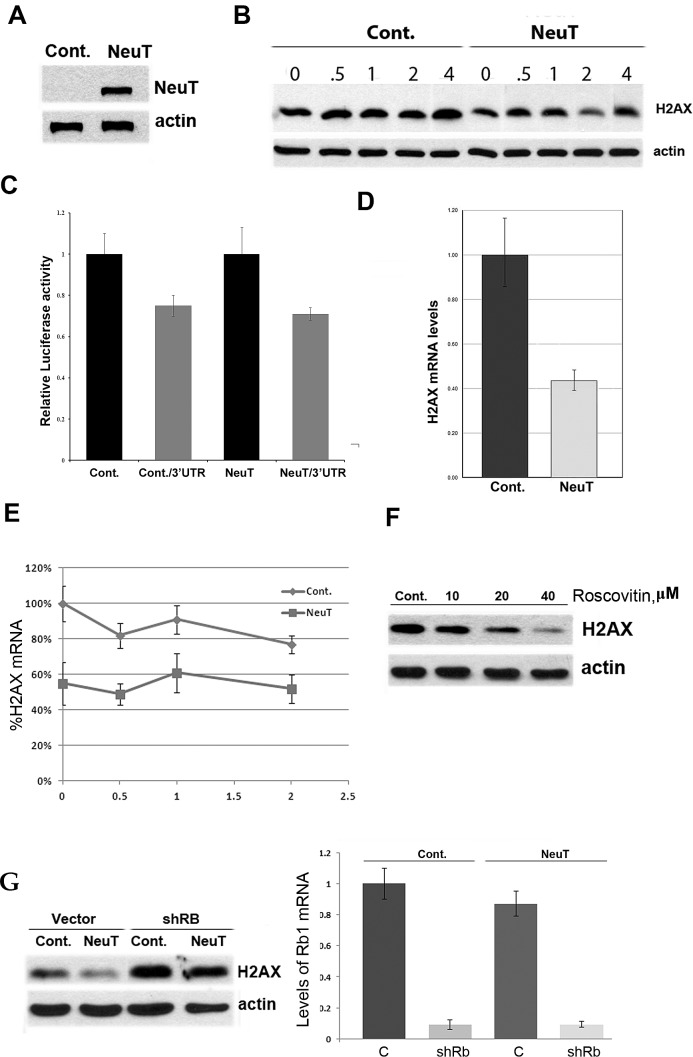
NeuT oncogene suppresses expression of H2AX A Expression of NeuT in MCF10A cells. MCF10A cells were infected with NeuT- expressing virus and two days post-infection selected with puromicin (0.75μg/ml). Control cells were infected with a corresponding empty virus. After 6 days of selection, cells were harvested, lysed, and analyzed by immunoblotting. B. NeuT expression does not affect stability of H2AX. MCF10A cells were infected as above. Cells were treated with emetine (10μM) and levels of H2AX at corresponding time points were assayed by immunoblotting. C. NeuT expression doesn't suppress translation of H2AX mRNA. MCF10A cells were infected as above. On day 6, post-infection cells were transfected with plasmid expressing renilla luciferase under H2AX 3'UTR control and the firefly luciferase used as an internal control. Translational efficiency was measured as renilla/luciferase ration (relative units). D. NeuT oncogene suppresses transcription of H2AX mRNA. MCF10A cells were infected as above, and Q-PCR was performed as described in Materials and Methods to measure H2AX mRNA. Each sample was analyzed in triplicates. E. NeuT expression does not affect stability of H2AX mRNA. MCF10A cells were infected as above. Cells were treated with actinomycin D (7μg/ml) and samples for RNA extraction were collected at corresponding time points. Q-PCR was performed as described in Materials and Methods. Each point represents three independent experiments. F. Inhibition of CDKs suppresses expression of H2AX. MCF10A cells were treated with indicated doses of roscovitine overnight. Cells were lysed and H2AX levels were assayed by immunoblotting. G. Rb silencing reverses suppression of H2AX levels caused by expression of NeuT. MCF10A cells were simultaneously infected with NeuT- expressing retrovirus (under blasticidin selection) and siRB1-expressing lentivirus (under puromycin selection); control cells were infected with corresponding empty viruses. Two days post-infection puromycin (0.75μg/ml) and blastocidin (10μg/ml) were added and after another 7 days cells were harvested, lysed, and analyzed by immunoblotting to assay H2AX levels (left panel) or by Q-PCR to assay level of Rb1 silencing (right panel).

While the mechanism of upregulation of p21 by Her2 in breast epithelial cells has been dissected previously, and involves the inflammatory loop and transcription factor Stat3 [23], the mechanism of H2AX downregulation by p21 remains unexplored. Therefore we next tested whether the changes in H2AX levels are caused by changes in protein degradation, translational efficiency, mRNA decay, or transcription. First, immortal human breast epithelial MCF10A cells were infected with control or Her2-expressing retrovirus, as in our prior publications (1), and Her2 expression was confirmed by immunoblotting (Fig. 2A). At day 5 post infection, after brief puromycin selection, the rate of degradation of H2AX in parental and MCF10A/Her2 cells was measured by immunoblotting following inhibition of translation by emetine. No significant degradation of H2AX either in control or NeuT-expressing cells was observed within six hours (Fig. 2B), indicating that the protein is relatively stable, thus eliminating the possibility that downregulation occurs via faster protein degradation. Next, to check if suppression of H2AX is due to less efficient translation, we used a 3'UTR Renilla/Luciferase double reporter (see Materials and Methods). In this reporter system Renilla is expressed under the control of 3'UTR of H2AX mRNA, while liciferase is extressed from the same plasmid without this 3'UTR, and serves as a standard for normalization. We observed minor translational suppression when H2AX 3'UTR was attached to the Renila gene; however, there was no difference in levels of the reporter luciferase/3'UTR between MCF10A and MCF10A/NeuT cells (Fig. 2C). Therefore, translation cannot explain downregulation of H2AX expression by Her2. Next, we compared the levels of H2AX mRNA in parental and MCF10A/NeuT cells by RT-PCR. As seen in Fig. 2D, NeuT expression causes H2AX mRNA levels to reduce by about 50%. To understand whether the effect was due to lower transcription or higher mRNA degradation, we blocked transcription with actinomycin D and monitored levels of H2AX mRNA at different time points (Fig. 2E). In both MCF10A and MCF10A/NeuT cells, the rates of H2AX mRNA degradation were similar, indicating that Her2 does not affect the RNA degradation. Therefore regulation of H2AX expression by NeuT occurs at the transcription level.

The main mechanism by which p21 may inhibit the transcription of a specific gene involves inhibition of Rb phosphorylation by CDK4, 6. This subsequently inactivates the E2F family transcription factors [24]. To test whether this pathway is involved in H2AX regulation, we inhibited CDK proteins to see if this would mimic the effects of p21 on H2AX expression. Accordingly, MCF10A cells were treated with a broad-specificity CDK inhibitor, roscovitine, and then levels of H2AX were monitored 15h later by immunoblotting. As seen in Fig. 2F, roscovitine treatment led to a dose-dependent suppression of H2AX expression. We further tested if knockdown of Rb1 reverses the effects of Her2 on H2AX downregulation. Using a lentivirus expressing Rb1-interfering shRNA that could be selected for using puromycin, we subcloned NeuT into a pCXbs retrovirus that was selected for using blastocidin. We titrated this virus to achieve levels of NeuT expression that were comparable with pBABE-based expression levels of Her2 and then verified that both viruses lead to similar (~70%) suppression of H2AX expression (data not shown). Next, MCF10A cells were simultaneously infected with lentivirus expressing shRb1 and retrovirus expressing NeuT. Corresponding ‘empty’ viruses were used as controls. As seen on Fig. 2G, 80% suppression of Rb1 almost completely reversed the effects of NeuT on H2AX downregulation, thus indicating that Rb1 is both necessary and sufficient for H2AX regulation. Therefore, transcriptional regulation of the DNA repair factor H2AX upon expression of the Her2/NeuT oncogene appears to involve the classical p21-CDK-Rb pathway.

We further investigated whether NeuT expression affects other components of the double strand DNA repair pathways, including the Homologous Recombination repair (HR) pathway and the Non-Homologous End Joining (NHEJ) pathway. Accordingly, we chose several major components of these pathways and tested their levels upon expression of NeuT by Q-PCR. Indeed, levels of components of the HR pathway, RPA1 and BRCA1, were significantly reduced in NeuT-expressing cells, while levels of BRCA2 and Rad51 remained almost unchanged. Similarly, levels of Ku70 and XRCC4 components of the NHEJ pathway declined upon NeuT transformation, while levels of Artemis, DNA PKCcs, and Ligase 4 did not change (Table 1). In addition, the levels of upstream sensor of double DNA breaks, MRE11a was also strongly suppressed. Therefore, it appears that expression of the Her2/NeuT oncogenes reduces expression of components of multiple DNA repair pathways. This may significantly jeopardize DNA repair and enhance DNA instability.

**Table:** Table1

	% suppression in Her2-expressing cells
H2AX	50% +/- 4%
Upstream sensor MRE11a	40% +/- 5%
HR BRCA1 BRCA2 Rad51 RPA	40% +/-3% 30% +/-6% 30% +/- 2% 65% +/- 4%
NHEJ Ku70 XRCC4 Artemis DNA PKcs Lig 4	50% +/- 6% 40% +/-2.5% 0% 0% 0%

### Her2/NeuT oncogene suppresses DNA repair

**Fig. 3:**
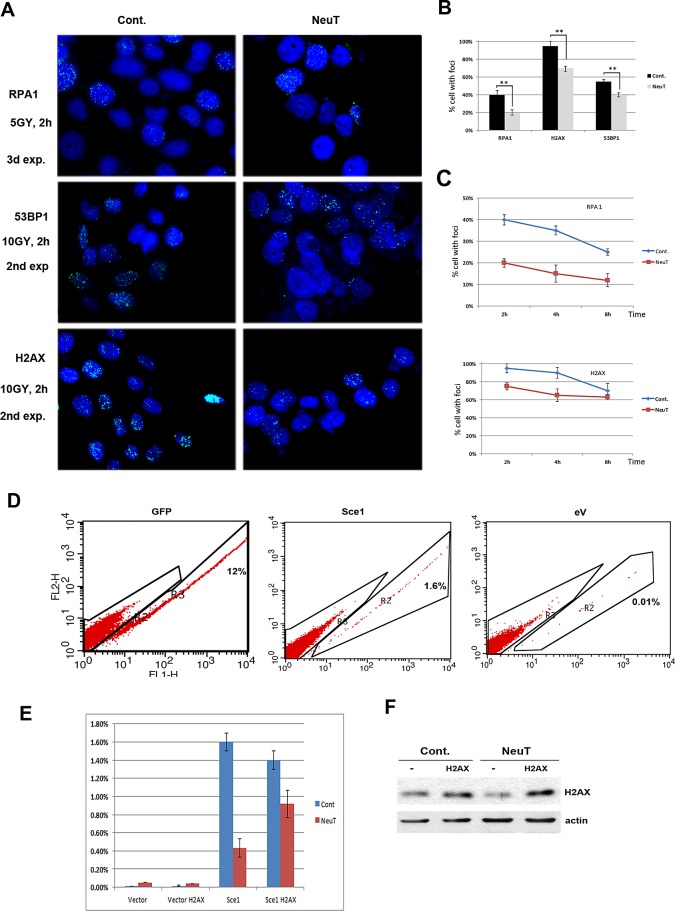
Her2 expression hampers DNA Damage response A-B. Formation of irradiation-induced foci is less efficient in MCF10A cells expressing Her2. MCF10A cells were infected as in Fig. 2 A. 6 days post selection, cells were 5Gy gamma irradiated, recovered for 2 hours and processed for immunofluorecence as described in Materials and Method. Quantification of foci-positive cells (C) was done by observing 300 nuclei. Experiment was repeated twice. C. Foci resolution is slower in MCF10A cells expressing Her2. Cells were treated as above and recovered for indicated period of time. Number of foci-positive cells was calculated after counting 300 nuclei. Experiment was repeated twice. D-F. H2AX rescues Homologous Recombination (HR) impaired by Her2 expression. D. MCF10 cells stably expressing HR reporter plasmid, DR-GFP, were infected with Her2-expressing retrovirus along with H2AX-expressing lentivirus. After 6 day selection, cells were transfected with a plasmid encoding either GFP, as a positive control (left panel), Sce1 endonuclease that produces double stranded breaks (middle panel) or empty vector as a negative control (right panel); 24 hr. later HR was assayed by FACS to detect number of cells that reconstituted functional GFP (see Materials and Methods for details). E. Quantification of experimental data shown in panel D. F. Levels of H2AX in MCF10A cells stably expressing HR reporter, DR-GFP and Her2. MCF10A cells from Fig. 3 D, E were lysed and analyzed by immunoblotting.

Upon exposure to genotoxic agents that cause double stranded DNA breaks, such as γ-irradiation, H2AX undergoes phosphorylation at Ser139. Phosphorylated H2AX (γH2AX) then recruits critical components of the DNA repair machinery leading to formation of ‘irradiation foci’ at the sites of DNA damage. Accordingly, downregulation of H2AX and other components of the DNA repair pathways upon expression of Her2 may lead to defects in DNA repair. To test this possibility, NeuT-transformed MCF10A cells were subjected to 5Gy gamma-irradiation while the formation of foci by γH2AX and the quantities of two other important DNA repair factors, RPA and p53BP1 were monitored by immunoflourescence. The resolution of foci, reflecting completed DNA repair, was also monitored at different time points after irradiation. For all three repair factors, γH2AX, RPA and p53BP1, we observed significantly reduced foci formation at the initial 2-hour time point (Fig. 3A, B). Interestingly, with γH2AX and RPA factors, we also observed slower foci resolution, which suggests suppressed DNA repair (Fig. 3C).

To investigate the effects of Her2 on the double strand DNA break repair by an independent method, we utilized a DR-GFP reporter system [25]. In this system, a GFP gene rendered un-functional by insertion of a recognition site for the I-SceI endonuclease can be repaired through homologous recombination using another non-functional GFP gene expressed from the same plasmid. As seen on Figs. 3D, E, expression of NeuT led to almost 75% reduction in the number of GFP positive cells, indicating that NeuT suppresses the HR DNA repair process. To test whether H2AX insufficiency is the sole cause of the reduced DNA repair capacity, we expressed H2AX in these cells using a lentiviral expression system (Fig. 3F). As seen in Fig. 3E, expression of H2AX significantly alleviated Her2-mediated suppression of HR. Therefore, H2AX appears to be the limiting factor in the DNA repair defects caused by Her2 expression. Overall, these independent experiments indicate that Her2 expression leads to defects in the double strand breaks DNA repair, and that the defect is associated with H2AX downregulation.

### Her2/NeuT oncogene increases DNA instability

**Fig. 4:**
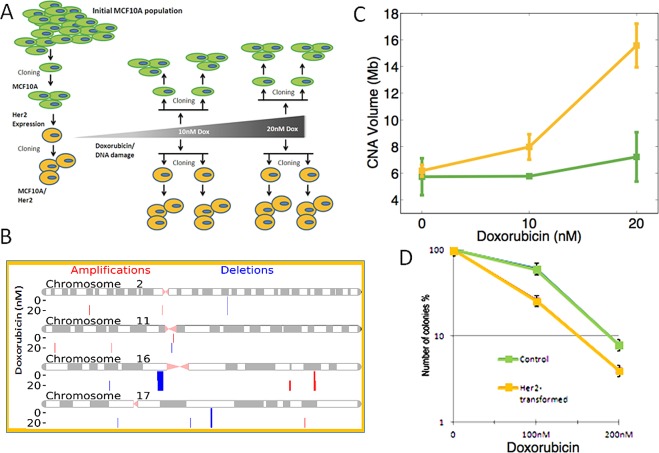
NeuT expression promotes DNA instability (A) Scheme of the experiment. See the Results section for explanations. (B) SMNA in MCF10A cells according to the SNP analysis. Deletions and amplifications along the chromosomes 2, 11, 14 and 22 are shown as example. (C) Quantification of SNMA in control and NeuT-expressing cells treated with indicated doses of doxorubicin. (D) Sensitivity of control and NeuT-expressing MCF10A cells to doxorubicin. Clonogenic assay was performed, as described in Materials and Methods.

Cell's reduced ability to repair double strand DNA breaks upon transformation with the Her2 oncogene may lead to DNA instability. To test this possibility directly, we characterized the level of spontaneous and induced DNA instability in Her2/NeuT-transfromed cells as compared to a parental control cells. Population of cells that persist in culture over long time periods may acquire DNA rearrangements and, thus, become genetically heterogeneous. To accurately assess the extent of DNA instability using SNP-arrays, we needed genetically homogenous populations, which were attained by growing clones of the MCF10A cells (Fig. 4A). Randomly selected clones were expanded, infected with retrovirus expressing NeuT (as in our prior experiments), and cloned again to yield three independent Her2/NeuT-transformed clones. We then used standard procedures to detect genomic amplifications and deletions (SCNA) by hybridization isolated genomic DNA to human SNP-arrays (See Methods and Materials). To characterize DNA instability, we normalized and segmented SNP-array data to detect individual alterations, their length and copy-number (Fig. 4B). We then identified a few SCNAs present in parental MCF10A cells that were probably acquired during immortalization (Fig. 4C). These parental SCNAs were removed from our analysis of DNA instability. We then used the total volume of all SCNAs, i.e. the sum of each SCNA's copy-number change multiplied by its length, as an aggregate measure of DNA instability (SI). First, we note that NeuT-transformed cells did not show a significantly higher load of SCNAs than parental populations (Fig. 4C) eliminating the possibility that the Her2/NeuT oncogene transformation by itself causes significant spontaneous DNA instability.

Next, we tested whether Her2-transformed cells exhibit increased level of induced DNA instability by exposing both a parental MCF10A clone and one of the MCF10A/NeuT clones to low-dose (sub-therapeutic) doxorubicin, thereby introducing DNA damage. To accomplish this, cells were treated overnight with 10 and 20nM of doxorubicin, then grown in normal media for 4 days and then cloned. DNA from these clones was isolated and hybridized on SNP-arrays. As seen on Fig. 4C, treatment with either 10nM or 20nM of doxorubicin did not cause significant DNA instability in parental cells; however, even low 10nM doses of doxorubicin triggered significant DNA instability in Her2-expressing cells, while 20nM caused even stronger effects (Fig. 4C). Therefore, overall expression of the Her2 oncogene in breast epithelial cells reduces the expression of major DNA repair factors, which leads to reduced DNA repair capacity and enhanced DNA instability.

In line with these results, we found that Her2-transformed MCF10A cells were significantly more sensitive (determined by colony survival) to doxorubicin treatment than the parental untransformed MCF10A cells (Fig. 4D). Therefore, the overall expression of Her2/NeuT oncogene in breast epithelial cells reduces the expression of major DNA repair factors, and leads to a reduced ability to repair DNA and enhanced DNA instability, which in turn affect cells’ sensitivity to DNA-damaging treatments.

## DISCUSSION

DNA instability is found in almost all human cancers. In some cancers, e.g. triple-negative breast cancer, DNA instability is an integral part of the process of tumorigenesis, and is driven by defects in DNA repair systems (e.g. mutations in BRCA1 or BRCA2), which result in further cancer-driving mutations. In other cancers, DNA instability results from defects in mitosis, which leads to large chromosomal rearrangements and aneuploidy. Yet, for the majority of cancers the origin of DNA instability is unclear. The major question in the field is whether DNA instability is the cause or the consequence of cancer.

Here, we investigated the origin of DNA instability in the model of Her2-positive cancer, where no obvious link to defects in DNA repair systems has been reported. We built upon our prior data that expression of Her2/NeuT oncogene in the breast epithelial cells MCF10A caused downregulation of the major DNA repair factor histone H2AX [10]. We then hypothesized that DNA instability in Her2-positive cancers results directly from the Her2 oncogene's activity. Since Her2 is expressed in early lesions (i.e. ductal carcinoma *in situ* (DCIS)), our hypothesis predicts that these lesions should exhibit DNA instability. Indeed, in human cancer studies, DNA instability was reported in early lesions, as early as DCIS [26-28]. Moreover, our hypothesis predicts that instability results from defects in DNA repair (specifically p21 mediated H2AX downregulation), and not from mitotic defects. In this study of DCIS lesions, DNA instability was monitored using the microsatellite instability assay. This assay should only detect focal SCNAs, and not than large chromosomal rearrangements [6]. Hence, it suggests that DNA instability in these lesions is indeed a result of improper DNA repair. Most convincingly, DNA instability in DCIS correlated with increased expression of Her2, upregulation of p21 and pleomorphic nuclei [29-31]. All these findings are highly consistent with our hypothesis that activation of Her2 upregulates p21, which downregulates histone H2AX, which induces DNA instability.

The data reported here provided direct experimental prove that expression of the Her2 oncogene alone in immortalized breast epithelial cells is sufficient to cause defects in the HR DNA repair pathway. Interestingly, expression of Her2 causes significant downregulation of multiple DNA repair factors besides H2AX. These factors are involved in both HR and NHEJ DNA repair pathways, and include RPA1, BRCA1, Ku70, XRCC4 and MRE11a. Therefore, it is possible that both pathways of double strand DNA break repair are jeopardized in Her2-expressing cells. Consistent with these findings, these cells had significantly increased vulnerability to genotoxic stress, as judged by a colony-forming assay. Suppression of DNA repair significantly increased DNA instability upon treatments with very low doses of doxorubicin. Indeed, incubation with 10 or 20nM doxirubicin created almost no SCNAs in control cells, yet many SCNAs in Her2-expressing cells.

In our prior study, we linked downregulation of H2AX upon expression of Her2 to activation of senescence signaling (i.e. induction of p21) and progression to a SWING state [10]. This dependence was shown in cultured cells. Here, we demonstrated that this pathway functions in vivo in a mouse model of Her2-positive cancer. Indeed, expression of Her2 in the mammary tissue led to induction of p21 and downregulation of H2AX. Furthermore, downregulation of H2AX was effectively reversed by the p21 knockout.

Further question addressed in this study is how Her2 affects expression of H2AX. While the pathway that links Her2 and p21 has been established and involves the transcription factor Stat3, the downstream section of the pathway, i.e. how p21 regulates expression of H2AX, has not been known. Here, we demonstrated that the major p21-controlled Rb pathway is involved in this regulation. Indeed, depletion of Rb reversed the effects of Her2 expression on H2AX levels. Furthermore, overexpression of Rb mimicked the effects of p21 expression on the levels of H2AX. Inhibition of CDKs had a similar effect, indicating that this regulation involves the p21-CDK-Rb pathway. Based on these findings, it is likely that this regulation also involves one or more of the eight E2F transcription factors.

Overall, this work describes a new mechanism of DNA instability in cancer. It demonstrates that senescence signaling, activated by Her2, leads to suppression of DNA repair pathways, increases DNA instability and increases vulnerability to genotoxic stresses.

## METHODS

### Cell cultures, treatments, and reagents

HEK293 and MCF10A cells were obtained from the American Type Culture Collection. MCF10A/DR-GFP stable cell line was obtained by transfection of parental MCF10A cells using GeneCellin (BioCellChalange, Toulon Cedex9, France) with a DR-GFP reporter vector, followed by 14 days of selection with puromycin. The DR-GFP reporter vector and plasmid expressing Sce1 nuclease were a kind gift from Dr. Powell, Memorial Sloan Kettering Cancer Center, New York.

HEK293 cells were grown in DMEM supplemented with 10% fetal bovine serum; MCF10A cells were grown in DMEM/ F12 (1:1, v:v) containing 5% horse serum and supplemented with 10 μg/mL insulin, 20 ng/mL epidermal growth factor, 100ng/mL cholera toxin, 0.5 μg/mL hydrocortisone, 100 units/ mL penicillin, and 0.1 μg/mL streptomycin (10A complete media); MCF10A-DR-GFP were grown in the same 10A complete media supplemented with puromycin (0.75 μg/mL). All cells were grown in a humidified environment at 37°C with 5% CO_2_.

Doxorubicin (Cat. #GR-319); puromycin (Cat. #GR-312), and roscovitine (Cat. #cc-205) were from BioMol (Plymouth Meeting, PA, USA); blasticidin was from InvivoGen (San Diego, CA; cat. #ant-bl-1); emetine (Cat. #E 2375) and actinomicin D (Cat. #A1410) were from Sigma (St. Louis, MO).

γ-IR spectroscopy of cells was performed using ^137^Cs source (GammaCell 40) at 64 rad/min.

### Recombinant retro- and lentiviral vectors

H2AX-expressing lentivirus and control ‘empty’ lentivirus were a kind gift from Dr. E. Brown (University of Pennsylvania Medical School, Philadelphia, PA); NeuT and control (pBABE) retroviral vectors were a kind gift from Dr. C. Spangenberg (Trost et al., 2005). This version of NeuT carries the activating V664E mutation (NeuT). Retrovirus expressing NeuT under blastocidine selection was obtained by subcloning NeuT from pBABE vector into pCXbsr retroviral vector. Cloning was done by Nahum Meller Custom DNA Constructs, 2340 Canterbury Rd, University Heights, OH, 44118. pCXbs was used as an ‘empty’ vector control. Lentivirus interfering with Rb1 was a kind gift from Professor Jim Xia (Boston University, Boston, MA).

Retro- and lentiviruses were produced as reported before [32, 33]. Briefly, HEK293T cells were co-transfected with plasmids expressing retroviral proteins Gag-Pol, vesicular stomatitis virus glycoprotein pseudotype and enhanced green fluorescent protein or our constructs using Lipofectamine 2000 (Invitrogen, Carlsbad, CA, USA). At 48 h after transfection, supernatants containing the retroviral particles were collected, aliquoted, and frozen at 80^0^ C until use. Cells were infected with diluted supernatant in the presence of 10 μg/mL Polybrene overnight, and were selected with puromycin (0.75 μg/mL) or blasticidin (10μg/mL) 48 h after infection. Retroviral vectors expressing enhanced green fluorescent protein was used as infection efficiency indicator: virus dilution was used to achieve approximately 90% of infected cells being GFP positive 2 days after infection. Lentiviruses were produced in the same manner except for HEK293T transfections, where lentivirus-specific packaging plasmids psPAX2 and PMD2.G from Addgene were used.

### Immunoblotting

Cells lysates were prepared as described previously [32]. Antibodies used in the study were β-actin from Sigma; NeuT (Ser 1981) were a kind gift from Dr. T Kowalik (University of Massachusetts Medical Center, Worchester); γH2AX (Ser139) were from Millipore (Cat.# 05-636; Billerica, MA, USA). Quantification of blots was performed using Quantity One software (Bio-Rad, Waltham, MA, USA).

### Immunohistochemistry

Mammary glands were excised from animals, spread on a Whatman filter strips, fixed in 4% formalin/PBS solution at 4^o^ C for 48h upon which tissue was transferred to 70% ethanol. Samples were embedded into paraffin and processed into slides (5 μm in thickness). H2AX and p21 staining was performed on these slides via standard ABC method (Vector Laboratory, Burlingame, CA, USA) using the following antibodies: p21^Waf-1^ Ab-9 (NeoMarkers, RB-032-P1; 1:25); and H2AX (Proteintech Group # 10856-1-AP; 1:100).

### Immunofluorescence

After treatments, cells were fixed in 100% ice-cold methanol/ acetone (50:50) at -20°C for 10 min, then permeabilized in 0.2% Triton X-100 / PBS for 15 min at room temperature (RT), then blocked in 3% BSA / PBS for 1 h, and then incubated with corresponding primary Ab at 4^o^C (γH2AX[Ser139](1:400); Cat. #05-636, Millipore, Billerica, MA, USA; 53BP1(1:50); BD Biosciences, RPA1(1:50); Calbiochem, Cat. #NA-18). Cells were washed out 3 times for 5 minutes each with 3% BSA / PBS followed by incubation with secondary Ab for 1 hour at RT. All antibody dilutions were made in Amplifying Antibody Dilution Buffer (ProHisto, Cat. #AA3; Columbia, SC). For RPA1 foci, cells were pre-extracted for 20 seconds with 0.2% Triton X-100 / PBS prior to fixation. Images were analyzed using Zeus fluorescent microscope (Carl Zeiss, Oberkochen, Germany).

### Mouse breeding

All animals were ordered from Jackson laboratories. FVB/N-Tg(MMTVneu)202Mul/J [Neu^+/+^;p21^+/+^] (F) was crossed with B6129SF2/J B6;129S2-Cdkn1a<tm1Tyj>/J p21^-/-^ (M); P1 females(Neu^+/-^; p21^+/-^) were back-crossed with B6129SF2/J B6;129S2-Cdkn1a<tm1Tyj>/J p21^-/-^ (M) and P2 females from the corresponding litter were genotyped (see below) to identify [Neu^+/-^; p21^-/-^] animals. Next, P2 (F) [Neu^+/-^; p21^-/-^] were back-crossed with B6129SF2/J B6;129S2-Cdkn1a<tm1Tyj>/J p21^-/-^ (M); then the female litter was genotyped and [Neu^+/-^; p21^-/-^] animals were used in further experiments. In parallel a FVB/N-Tg(MMTVneu)202Mul/J [NeuT^+/+^;p21^+/+^] (F) was crossed with a p21WT parent M B6129SF2/J (M). The female litter [Neu^+/-^; p21^+/+^] was used as a control. FVB/NJ females were used as a control for FVB/N-Tg(MMTVneu)202Mul/J mice.

Animal maintenance and experiments were conducted in compliance with the guidelines of the Institutional Animal Care and Use Committee.

### Genotyping

For genotyping, genomic DNA was purified from tails using Qiagen DNeasy Blood and Tissue kit (Cat. #69504; Valencia, CA). PCR to define p21 and NeuT status was performed according Jackson Laboratories recommendations.

### Real Time PCR analysis

Total RNA was isolated from cells using Qiagen RNeasy Mini kit (Cat. #74104; Valencia, CA;). The RETROscript Kit (Ambion, Austin, TX, USA; Cat. #AM1710) was used to convert 2mg RNA into cDNA. qRT–PCR was performed using the SensiFast SYBR Green PCR kit (Bioline, Cat. # Bio-92020; London,UK;) according to the manufacture's protocols; expression levels of GADPH were used as an internal control. Real-time analysis was implemented with a Applied Biosystems S7300 instrument using the following primers:

H2AX: from Qiagen (Cat.#QT00233023; Valencia, CA)

GAPDH:Forward: 5' GAA GGT CGG AGT CAA CGG ATT T Reverse: 5' ATG GGT GGA ATC ATA TTG GAA

Rb1:

Forward: 5'CTCTCGTCAGGCTTGAGTTTG Reverse: 5' GACATCTCATCTAGGTCAACTGC

BRCA1:

Forward: 5' TCCCATCTGTCTGGAGTTGA Reverse: 5' TGTGAAGGCCCTTTCTTCTG

BRCA2:

Forward: 5' TGCCTGAAAACCAGATGACTATC Reverse: 5' AGGCCAGCAAACTTCCGTTTA

MRE11a:

Forward 5' ATCGGCCTGTCCAGTTTGAAA Reverse 5' TGCCATCTTGATAGTTCACCCAT

XRCC4:

Forward: 5' TGCAAAGAAATCTTGGGACAG Reverse: 5' TGCTCCTTTTTCGACGTCTC

Ligase 4:

Forward: 5' AGCAAAAGTGGCTTATACGGATG Reverse: 5' TGAGTCCTACAGAAGGATCATGC

Artemis:

Forward: 5' CCAAAGTACGGAGCCAAAGT Reverse: 5' TTGGCAGAGGATCATCAAAG

DNA-PKcs:

Forward: 5' AGCTGGCTTGCGCCTATTT Reverse: 5' GGGCACACCACTTTAACAAGAC

Ku70:

Forward: 5' GCTAGAAGACCTGTTGCGGAA Reverse: 5' TGTTGAGCTTCAGCTTTAACCTG

RPA1:

Forward: 5' CCGTAGTAATGGGACGGATG Reverse: 5' GCAGAAGGGGGATACAAACA

qPCR results are summarized as mean ± standard deviation.

### Clonogenic assay

Cells after treatments were counted using Scepter (Cat. #PHCC00000, Millipore, Billerica, MA, USA) and plated in triplicates on 100-mm Petri dishes. After 10 days, the colonies were stained with 0.5% crystal violet in 70% ethanol and quantified. Survival was calculated as a percentage of colonies formed relative to untreated controls.

### 3'UTR translation assay

MCF10A cells were infected with “empty” or NeuT expressing retrovirus and selected with puromycin. On day 6 post-infection, cells were transfected using GeneCellin (BioCellChalange) with psiCHECK2 (Promega) vector (1μg per well in 6-well plate) containing the 3' UTR of H2AX cloned in the multiple cloning site of *Renilla* (kind gift from Dr. Chowdhury, Harvard Medical School.) After 24 h we measured renilla and luciferase activities using the Dual Luciferase Assay System (Promega) and the TopCount NXT microplate reader (Perkin Elmer), as per the manufacturer's instructions. Data were normalized to Firefly luciferase. Each measurement was done in triplicates.

### Measurement of HR activity with DR-GFP assay

HR activity was evaluated using the DR-GFP recombination substrate with a recognition site for the I-SceI endonuclease in one copy of the GFP gene. A gene conversion event within this substrate results in the expression of intact GFP protein [34]. Exponentially growing MCF10A/DR-GFP cells infected with corresponding viruses were transfected with a plasmid expressing the I-SceI endonuclease or empty vector. After growth for an additional 24 h in fresh media, cells were harvested by trypsinization and re-suspended in PBS. Cells were analyzed for GFP expression by flowcytometry.

### FACS analysis

FACS analysis was performed on BD Biosciences (San Jose, CA, USA) FACSCalibur, and data were analyzed with BD FlowJo (Tree Star, Inc.;10.0.7 software.)

### Assessment of Somatic Copy Number Alterations (SCNA)

DNA was isolated from individual clones and genotyped using Affymetrix Genome-wide Human SNP Array 6.0 (Cat. #901150) courtesy of the Broad Institute's Genome Sequencing and Analysis Program.

SNP probe intensities were quantified and DNA copy number was determined by segmenting SNP probes along the genome into tracks of equal copy number using the GLAD software package, an adaptive weights smoothing algorithm [35]. SCNAs were then identified from these tracks using a simplified calling rubric. Histograms of background-subtracted Log2 mean segment intensity were generated and then smoothed using a Gaussian kernel. Each sample's histogram exhibited 3-4 well-defined peaks, separated by approximately integer distances. Mean track intensities were scaled by a multiplicative constant such that the second peak, always the sample mode, was scaled to a ploidy value of 2.0 (diploid). All track were then assigned ploidy by rounding their intensity to the nearest integer, baring one exception. If a track (1) contained only 1-2 probes, (2) was flanked on the left and right by tracks with the same ploidy, and (3) was within 1.0 intensity of its flanking assignment, the track was merged with its flanking assignments (e.g. a very short track with a scaled signal intensity of 3.72, and neighboring tracks assigned a ploidy of 3, would be merged with its neighboring tracks to forge one long SCNA of ploidy 3, rather than rounded to a ploidy of 4. SCNA were then defined as uninterrupted tracks of ploidy other than diploid. We chose this method, rather an existing software package, because (i) our samples were clonal and did not need stromal rectifications, and (ii) we wanted an approach that did not bias towards specificity or sensitivity in calling SCNA, as we wanted to use these calls only to assess overall DNA instability.

Ancestral SCNA (existing in the cell lines prior to experimentation) were removed by identifying an ancestral genome. This ancestral genome was defined by SCNA that were shared (equal ploidy and ≥75% overlap) among all un-mutagenized (0 nM Dox) among all un-transformed (pBABE) MCF-10A clones. If a SCNA was shared (defined above) between a sample of interest and the ancestral genome, then it was excluded from our assessment of DNA instability. DNA instability was assessed by counting the total number of SCNA in a sample and by measuring their aggregate volume. Each SCNA individual volume was defined as its length (end location minus start location) multiplied by its amplitude (absolute difference from diploid).
